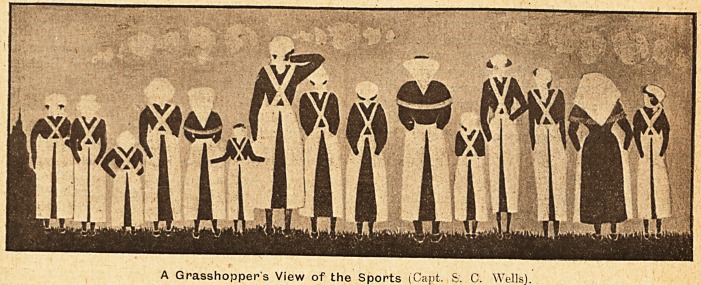# Nursing Progress and Its Developments: II. The Probationer as Student

**Published:** 1917-12-15

**Authors:** 


### THE MATRONS' AND SISTERS' DEPARTMENT.

A Grasshopper's View of the Sports (Capt. S. C. Wells).

### NURSING PROGRESS AND ITS DEVELOPMENTS.

### II. The Probationer as Student.

In the old-fashioned training-school the band of probationers were regarded as so much raw material to be utilised in carrying on the work of the hospital. By dint of performing their duties under the superintendence of women more skilled than themselves, their usefulness increased month by month. Incidentally, a little physiology was imparted, but the time needed to master its bare elements had to be saved out of scanty off-duty hours at the expense of exercise or sleep. Hurried lectures delivered by tired men to harassed and too often uncomprehending women formed a part of this system. Finally, after an easy-going examination, the probationer, unless remarkably unsatisfactory in one way or another, was certificated as a matter of course.

It must be definitely recognised that the time has gone by for regarding the probationer merely as a form of cheap labour for the hospitals. Every profession which values the status of its members is looking into the matter of training and taking steps to obviate the immense waste involved in a bad beginning. Under Mr. Fisher's new Education Bill no employer will be allowed to exploit youths of either sex for the performance of even the lowest forms of labour. The period of apprenticeship or training is to be markedly educational. A generous allowance of time is to be apportioned not only to learning the things which pertain to each calling, but to wider studies, and the hours devoted to mental improvement are to be chosen at a time of day when the faculties are fresh. If this principle be applied to the science and art of nursing it will be seen that it involves far-reaching changes in that system of training probationers which still lingers in many parts of the country.

The College of Nursing is fortunate in possessing among its promoters many women who have worked out this principle of the probationer as student and have translated it into action. It is to Miss Lloyd Still, whose name on the prospectus of the College of Nursing is one of its guarantees, that the initiative belongs of introducing the sister tutor into the training-school as an essential pivot round whom the entire course of instruction revolves. In smaller hospitals, it is true, the matron may herself be the best person to act as tutor. But there are few well-equipped training-schools in which the matron has sufficient time at her disposal for this important work. It is the duty of the sister tutor, working always, it goes without saying, in subordination to and in close conjunction with the matron, to direct the studies of every probationer, to assign her the necessary hours of study, to select her text-books, to encourage her to improve herself in any direction in which her education may be defective, to supervise her note-taking, and to check her impulse to cram her mind with ill-digested fragments of science "falsely so called." The good which results from the appointment of a highly-trained tutor to gather up the threads of the teaching and devote her entire time to seeing that the probationers learn aright is incalculable. It places the probationer at once in her proper place as a student. It does away with the haphazard effect of lectures and classes. It makes examinations a reality. It will soon be recognised as essential to the well-being of every institution undertaking to give training.

We welcome the recent introduction of the sister tutor into the Poor-Law infirmary. At the East Ham Infirmary, known as the Whip Hospital, the matron, who is an earnest adherent of the College of Nursing, has now appointed a skilled tutor to supervise the probationers' studies in well-appointed class-rooms—an example which, it may be safely prophesied, will speedily be followed elsewhere.

The College of Nursing has among its first aims that of ensuring that the probationer shall in the future be regarded by her employers as a student, first and foremost. The experience of every profession goes to show that under this system the practical abilities are raised, and that the value of the workers is immeasurably increased.

☛ The previous article appeared in "The Hospital" of December 8, 1917.

## Figures and Tables

**Figure f1:**